# Association Between Emphysema and Coronary Artery Calcium on Low-Dose CT in Urban Chinese Adults: Does Lifestyle Matter?

**DOI:** 10.3390/healthcare14060736

**Published:** 2026-03-13

**Authors:** Zhenhui Nie, Geertruida H. de Bock, Judith M. Vonk, Rozemarijn Vliegenthart, Xiaofei Yang, Matthijs Oudkerk, Xiaonan Cui, Zhaoxiang Ye, Monique D. Dorrius, Maaike de Vries

**Affiliations:** 1 Department of Epidemiology, University Medical Center Groningen, University of Groningen, 9700 RB Groningen, The Netherlands; z.nie@umcg.nl (Z.N.); g.h.de.bock@umcg.nl (G.H.d.B.);; 2 Groningen Research Institute for Asthma and COPD (GRIAC), University Medical Center Groningen, University of Groningen, 9700 RB Groningen, The Netherlands; 3 Department of Radiology, University Medical Center Groningen, University of Groningen, 9700 RB Groningen, The Netherlandsm.d.dorrius@umcg.nl (M.D.D.); 4 Faculty of Medical Sciences, University Medical Center Groningen, University of Groningen, 9700 RB Groningen, The Netherlands; 5 Department of Radiology, Tianjin Medical University Cancer Institute and Hospital, Tianjin 300060, China

**Keywords:** computed tomography, screening, coronary artery calcification, emphysema, cardiovascular disease, precision medicine

## Abstract

**Background and Objectives**: Emphysema and coronary artery calcium (CAC) share common lifestyle-related risk factors, yet their association in Chinese populations remains understudied. This study investigated how lifestyle factors influence the association between emphysema and CAC score in an urban Chinese general population. **Methods**: The study included 1000 participants from the Chinese Nelcin-B3 urban general population study originating in 2017 who underwent low-dose CT (LDCT) screening and comprehensive CT assessment. Emphysema was visually assessed by subtype and severity. CAC was measured using the Agatston method and categorized as 0, 1–100, and >100. Questionnaire-based lifestyle factors (smoking, BMI, diet, physical activity, alcohol consumption and environmental exposures) were categorized based on number of unfavorable behaviors. Multivariable multinomial logistic regression adjusted for age, sex, education and cardiovascular risk factors examined the associations between emphysema and CAC, with interactions and stratified analyses for lifestyle effects. **Results**: Emphysema was present in 62.3% of the participants, with centrilobular being the most common subtype (61.5%). Paraseptal emphysema was associated with both CAC 1–100 (OR: 2.07 [1.03–4.15]) and CAC > 100 (OR: 2.94 [1.26–6.84]). Severe emphysema was linked to CAC > 100 (OR: 3.50 [1.38–8.84]). These associations were stronger in the intermediate unhealthy lifestyle group for paraseptal (OR: 5.41 [1.70–17.22] and moderate and severe emphysema (OR: 9.64 [1.64–56.55]; OR: 3.73 [1.07–13.06]), respectively, but not significantly different. **Conclusions**: While paraseptal and severe emphysema are associated with higher CAC scores, there is no modifying effect of lifestyle factors. These findings suggest that cardiovascular risk assessment could be of importance in individuals with emphysema. Further longitudinal studies are needed to clarify the clinical implications.

## 1. Introduction

The “Big 3” (B3) diseases—lung cancer, coronary artery disease (CAD), and chronic obstructive pulmonary disease (COPD)—are major causes of mortality in China [1]. According to national Chinese studies conducted between 2014 and 2021, approximately 630,000 lung cancer deaths and 3.5 million cardiovascular deaths are reported annually, For COPD, it has been shown that it affects 13.6% of individuals older than 40, thereby significantly contributing significantly to the national disease burden [2,3,4]. COPD and CAD share chronic inflammation as a key pathological mechanism, suggesting a possible link between the extent of lung damage and coronary atherosclerosis [5,6,7]. Moreover, both diseases are influenced by several common risk factors such as aging, smoking, and exposure to air pollution. These may contribute to the development and progression of both conditions through systemic inflammation, oxidative stress, and endothelial dysfunction [6,7,8].

Low-dose computed tomography (LDCT) is widely used for lung cancer screening, offering the potential to diagnose lung cancer at an early stage and to reduce lung cancer-related mortality rates [8,9]. Beyond lung cancer detection, LDCT often reveals incidental findings such as emphysema, a hallmark of COPD, and coronary artery calcium (CAC), a reliable and non-invasive marker of atherosclerosis and future CAD events [5,10,11]. While some studies have examined the association between emphysema and CAC, particularly in Western populations, the findings remain inconsistent [10,12]. Evidence suggests the possibility of COPD-subtype-specific associations and variability across different study populations [10,12]. In addition, a healthy lifestyle is known to reduce the risk of cardiovascular disease (CVD) risk in patients with COPD [13], while unhealthy behaviors may elevate CVD risk through mechanisms such as systemic inflammation [14]. Given the close link between emphysema and systemic inflammation, a key driver of atherosclerosis, it is important to evaluate the association between emphysema and CAC across groups with varying lifestyle factors.

Previous studies have primarily focused on the direct relationship between emphysema and CAC, often without considering the potential influence of lifestyle factors. Evaluating whether this relationship persists after adjusting for lifestyle is essential, as it may point to other underlying mechanisms such as chronic inflammation or genetic predisposition. Moreover, most existing studies have investigated individual lifestyle components—such as diet, physical activity, and smoking on either emphysema or cardiovascular risk—in isolation, rather than examining their combined impact on emphysema or cardiovascular risk [14,15]. Evidence from Asian populations remains limited, despite notable differences in lifestyle and environmental exposures compared to Western populations [15,16].

In this context, our study aimed to investigate the association between emphysema and CAC score in an urban Chinese general population. Additionally, we examined how this association is impacted by lifestyle factors, providing insights into population-specific strategies for identifying high-risk individuals who may benefit from targeted screening.

## 2. Materials and Methods

### 2.1. Study Population

This study included data from the Nelcin-B3 study, a large population-based cohort in China with prospective participant inclusion [17]. The Nelcin-B3 study was a collaborative initiative between three large hospitals in China and an academic hospital in the Netherlands [18]. Its aim was to refine lung nodule management criteria for the Chinese population and enhance the effectiveness of LDCT screening for COPD and CVD [17]. The data for the present analysis were derived from the NELCIN-B3 cohort in Tianjin. From May 2017 to December 2019, participants were recruited by local media and inclusion criteria were checked by doctors in five community health service centers [19]. In total, 4000 asymptomatic participants were included, aged 40–74, who had lived in Tianjin for at least three years and had no history of lung cancer [17]. Participants who had received a chest CT scan within the last 12 months were excluded. At the baseline screening, participants underwent a chest LDCT scan and completed a questionnaire that included items assessing personal characteristics and lifestyle. The interval between the questionnaire completion and the LDCT examination did not exceed two months, to ensure accurate identification of potential CVDs and enhance the interpretation of CT findings. The study was approved by the ethics committee of Biomedicine Research of Second Military Medical University in China (registration number: NCT03992833) and all participants provided written informed consent.

Of the 4000 participants enrolled in the Nelcin-B3 cohort, comprehensive CT assessment was conducted in 1000 participants with consecutive scans (ID number 1000 to 2000) performed between May and August 2017. For the current analyses, these 1000 participants were selected. Of these 1000 participants, participants with incomplete data, improper CT reconstruction or having a history of coronary artery disease or stents were excluded, resulting in 871 participants included in the analysis. One participant aged 76 years underwent screening due to voluntary participation and met all other eligibility criteria; therefore, this single case was retained in the analysis.

### 2.2. CT Scan Acquisition

All participants underwent a non-ECG triggered LDCT examination with 64-detector row CT system (Somaton Definition AS 64, Siemens, Erlangen, Germany) at end-inspiratory breath hold [17]. Positioning of participants was headfirst, supine, and with arms above the head. Chest scan acquisition used the following parameters: 120 kVp, 35 mAs, pitch of 1.0, D45F were applied to reconstruct images at 1.0/0.7 mm thickness/increment for emphysema assessment, and B35F at 3.0/1.5 mm thickness/increment for CAC score assessment.

### 2.3. CT Parameter Assessment

An experienced radiologist, radiologist 1 (X.Y., with 5 years of experience), reviewed the CT scans for emphysema utilizing the Minimum Intensity Projection (MinIP) technique at lung window setting (window center: −750 HU, window width: 700 HU). Interobserver agreement was determined based on 100 randomly selected cases assessed by a second radiologist (Z.N., with 3 years of experience). Emphysema was evaluated with the presence of clearly defined or poorly defined areas of reduced attenuation or lucencies [20]. If at least trace was present, emphysema was further categorized as one of the three predominant subtypes of emphysema (centrilobular [CLE], paraseptal [PSE], and mixed type including panlobular). The severity of emphysema was categorized into mild, moderate, and severe using an adapted classification based on Fleischner criteria (Table 1) [20].

The CAC score was determined according to Agatston’s method by two experienced readers at lung window settings (window center: 50 HU, window width: 350 HU) using syngo.via software (VB30A, Siemens Healthineers, Erlangen Germany). This software was used to quantify the calcified areas and Agatston score was automatically calculated by multiplying the lesion area by a density weighting factor. Discrepancies were resolved by consensus. The CAC score was collected as a continuous variable and was, for the sake of the analysis, categorized into CAC score groups (0, 1–100, and >100) according to Agatston score thresholds [21].

### 2.4. Questionnaire Data and Definitions

Personal and lifestyle information was collected through the baseline questionnaire in 2017 (details in Supplementary Table S1). Personal characteristics included demographics (year of birth, sex, weight, height, and education level) and clinical risk factors such as self-reported diabetes, hypertension, and hyperlipidemia. Education level was categorized into low and high [22].

Based on previous studies and national guidelines for CVD prevention in China [23,24,25], six unhealthy lifestyle factors were defined based on questionnaires, including (1) current or former smoking; (2) being underweight (BMI < 18.5 kg/m^2^) or overweight (BMI ≥ 24.0 kg/m^2^); (3) having any of the following poor dietary habits: no daily intake of fresh fruits, no daily intake of vegetables, or daily consumption of red meat; (4) low or moderate physical activity level; (5) alcohol consumption more than three times per week; (6) toxic environmental exposure, defined as regular exposure to cooking fumes, living or working in buildings located along busy streets or contact with toxic or harmful substances for three months or longer. Participants were then grouped into three lifestyle groups, based on the number of these unfavorable lifestyle factors: favorable (0–1), intermediate (2–3) and unfavorable (4–6).

### 2.5. Statistical Methods

All statistical analyses were conducted using SPSS 28.0 (IBM Corporation, Armonk, New York 10504, NY, USA).

#### 2.5.1. Baseline Characteristics

Baseline characteristics and lifestyle factors of participants were described, summarized, and stratified by CAC score category. Differences in participant characteristics between the CAC score categories were tested with the chi-square test for categorical variables and with the Kruskal–Wallis H test for continuous variables.

#### 2.5.2. Interobserver Agreement

To assess consistency between readers, kappa statistics were calculated for the presence of emphysema.

#### 2.5.3. Directed Acyclic Graph (DAG)

DAG was developed to illustrate the hypothesized relationships between emphysema (exposure), CAC score (outcome), and potential confounders (Supplementary Figure S1). Age, sex, education level and cardiovascular risk factors were identified as key confounders, given their potential impact on both emphysema and CAC. Lifestyle factors (i.e., smoking, BMI, diet, physical activity, alcohol and environmental exposure) were also considered as potential confounders.

#### 2.5.4. Regression Analysis

Multivariable multinominal logistic regression analyses were used to identify the association between emphysema, emphysema type (CLE, PSE or mixed type) or severity (mild, moderate or severe) and CAC score categories, adjusted by: (1) age, sex, education level, diabetes, hypertension, hyperlipidemia; (2) age, sex, education level, diabetes, hypertension, hyperlipidemia and unfavorable lifestyles (smoking, unhealthy BMI, less healthy dietary habits, low/moderate physical activity, alcohol consumption and environmental exposure); (3) age, sex, education level, diabetes, hypertension, hyperlipidemia and number of unfavorable lifestyle factors.

To explore whether the association between emphysema, emphysema type (CLE, PSE or mixed type) or emphysema severity (mild, moderate or severe) and CAC score differed across subgroups defined by each lifestyle factor, stratified analyses were conducted and adjusted by age, sex, education level, diabetes, hypertension, hyperlipidemia and other lifestyle factors. Results were visualized using a heatmap. To assess if associations were significantly different between the healthy and unhealthy lifestyle strata, interaction analyses were performed by adding the interaction term to the respective unstratified regression models adjusted for age, sex, education level, diabetes, hypertension, hyperlipidemia and other lifestyles.

For all the analyses described above, odds ratios (ORs) and related 95% confidence intervals (95% CIs) were provided, and a *p*-value of <0.05 was considered statistically significant.

## 3. Results

### 3.1. Participant Characteristics

**Figure 1:**
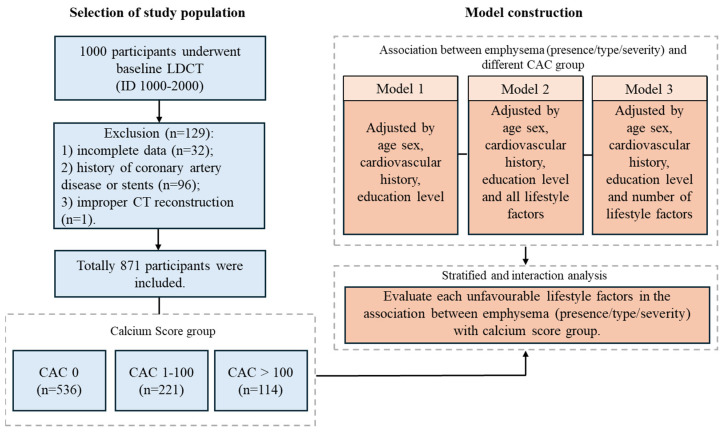
Flowchart of the study design.

Of the 1000 participants included in this study, 129 were excluded due to incomplete questionnaire data (n = 32), issues with chest CT reconstruction (n = 1) or history of coronary artery disease or stents (n = 96) (Figure 1). The study included 871 participants with a median age of 62 years (range: 40–76) at CT examination; 45.5% were male. Emphysema was present in 62.2% of participants, with the most common type being centrilobular emphysema (38.3%), followed by mixed type (17.9%) and paraseptal emphysema (6.0%) (Table 2). Among those with emphysema, 88.9% had mild emphysema, 4.2% had moderate emphysema, and 6.8% had severe emphysema. Out of all participants, 61.5% had no CAC score (n = 536), 25.4% had a CAC score between 1 and 100 (n = 221) and 13.1% had a CAC score > 100 (n = 114). Participants with higher CAC scores were older, more likely to be male, and have a low education level and significantly higher prevalence of emphysema, compared to participants with no CAC score. Having a history of diabetes and hypertension was more prevalent in participants with higher CAC scores.

Significant differences were observed in smoking and alcohol consumption across CAC groups (both *p* < 0.001) (Table 3). Participants with CAC > 100 were more frequently current/former smokers (58.8% vs. 24.8% in CAC 0) and alcohol consumers (32.5% vs. 16.8% in CAC 0). No significant differences between the CAC score categories were found for BMI, dietary habits, physical activity, or environmental exposure (*p* > 0.05). Finally, a higher number of unfavorable lifestyle factors was associated with higher CAC scores (*p* < 0.001). Specifically, 28.9% of the CAC > 100 group had 4–6 risk factors, compared to only 14.4% in the CAC 0 group.

### 3.2. Interobserver Agreement

Interobserver agreement of the two radiologists was 0.78 (95% CI: 0.75–0.82) for the presence of emphysema.

### 3.3. Association Between Emphysema and Calcium Score

**Figure 2:**
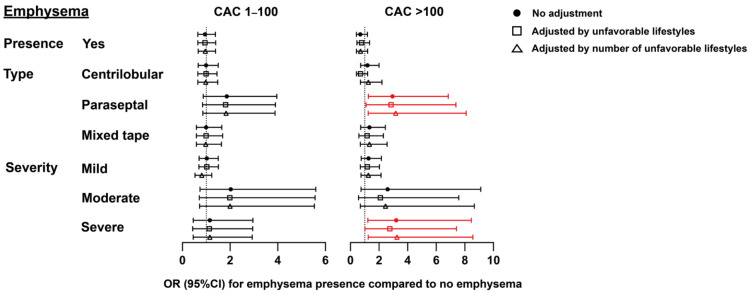
Associations between emphysema and CAC groups for presence of emphysema, emphysema type and emphysema severity. The models are adjusted for age, sex, education level and cardiovascular risk factors (i.e., diabetes, hypertension, hyperlipidemia). Red means the likelihood of increased CAC score; a CAC score of 0 was used as the reference.

The presence of emphysema was not significantly associated with CAC (Figure 2, Supplementary Table S2). Paraseptal emphysema showed a significant association with CAC > 100 (OR: 2.94 [1.26–6.84]), as did severe emphysema (OR: 3.26 [1.24–8.57]). After adjustment for unfavorable lifestyles (either as individual factors or cumulative number), the associations between paraseptal emphysema and severe emphysema with higher CAC scores remained significant (Figure 2, Supplementary Tables S3 and S4).

**Figure 3:**
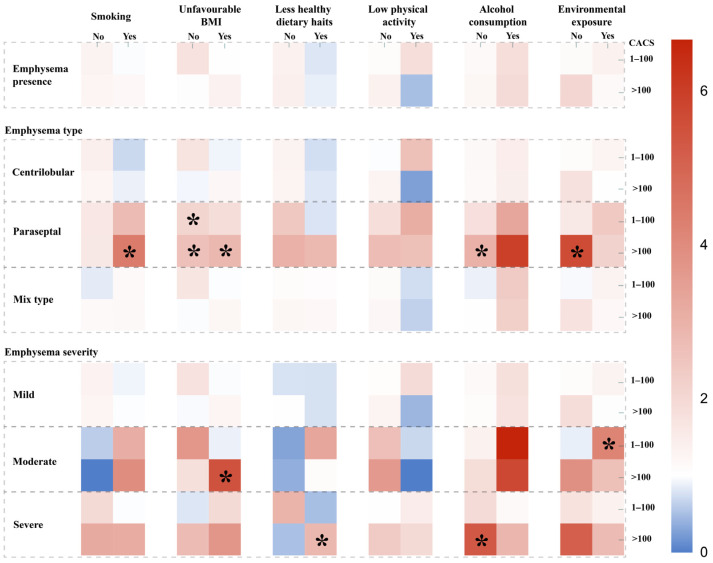
Heatmap of associations between emphysema presence/type/severity and CAC groups, stratified by unhealthy lifestyle factors. Each column represents a specific unhealthy lifestyle factor (dichotomized as “no” and “yes” groups), and each row corresponds to different patterns of emphysema (presence, type, severity). The models were adjusted for age, sex, education level, cardiovascular risk factors (diabetes, hypertension, hyperlipidemia), and other lifestyle factors. Color indicates the magnitude of odds ratios (ORs) for elevated CAC scores (reference: CAC = 0), with red representing higher and blue representing lower ORs; * indicates statistical significance at *p* < 0.05.

We stratified the data by lifestyle factors into two categories and assessed the differences between the presence, type and severity of emphysema and CACS in these strata (Figure 3). In none of the lifestyle strata was emphysema presence significantly associated with CAC score (Figure 3, Supplementary Table S5). However, paraseptal emphysema showed significant associations with higher CAC scores in specific groups: smokers (CAC > 100: OR 4.41 [1.16–16.78]), individuals with a healthy diet (CAC 1–100: OR 2.42 [1.00–5.83]; CAC > 100: OR 3.01 [1.00–9.04]), those with unhealthy diet (CAC > 100: OR 2.81 [1.08–7.30]), participants who did not consume alcohol (CAC > 100: OR 3.00 [1.01–8.94]) and those without toxic environmental exposure (CAC > 100: OR 5.63 [1.30–24.35]) (Figure 3, Supplementary Table S6). Severe emphysema was linked to CAC > 100 in those with healthy diet (OR 2.83 [1.05–7.67]) and in those without alcohol consumption (OR 5.30 [1.48–19.04]). Moderate emphysema was associated with CAC > 100 in those with unfavorable BMI (OR 5.46 [1.00–30.42]) and with CAC 1–100 in those with toxic environmental exposure (OR 4.18 [1.10–15.87]) (Figure 3, Supplementary Table S7). None of the unfavorable lifestyle factors significantly modified the associations between emphysema, emphysema type or emphysema severity and CAC score (Supplementary Tables S5–S8).

**Figure 4:**
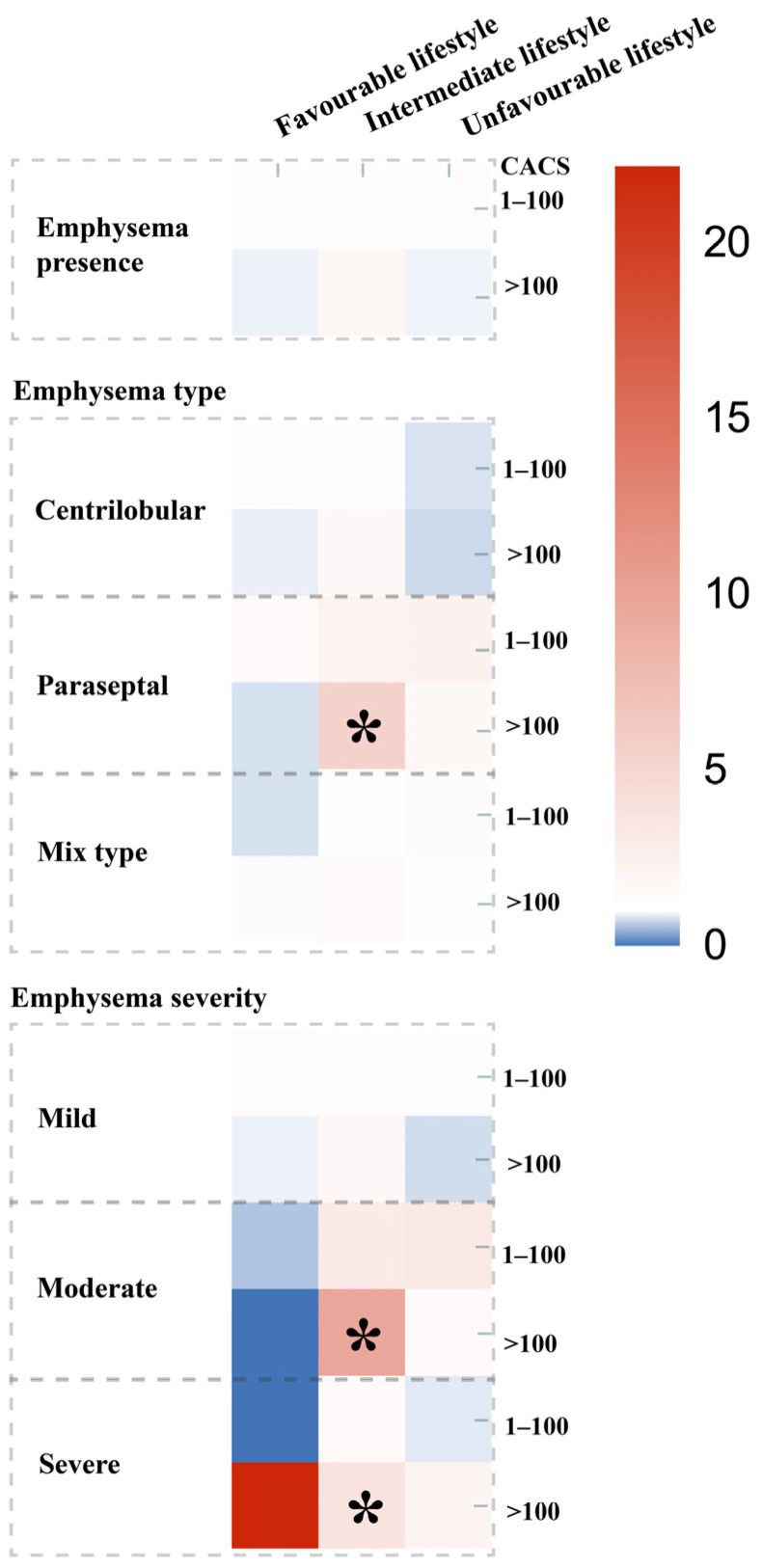
Heatmap of associations between emphysema and CAC groups stratified by the number of unhealthy lifestyle factors. Each column represents the number of unhealthy lifestyle factors, divided into favorable lifestyle, intermediate lifestyle and unfavorable lifestyle. Each row corresponds to different patterns of emphysema (presence, type, severity). The models were adjusted for age, sex, education level, cardiovascular risk factors (diabetes, hypertension, hyperlipidemia), and other lifestyle factors. Color indicates the magnitude of odds ratios (ORs) for elevated CAC scores (reference: CAC = 0), with red representing higher and blue representing lower ORs; * indicates statistical significance at *p* < 0.05.

Next, we divided the data by lifestyle groups and assessed the difference between presence, type and severity of emphysema and CACS in these groups (Figure 4). As shown in Figure 4, significant associations were found for paraseptal (OR 5.41 [1.70–17.22]), moderate (OR 9.64 [1.64–56.55]), and severe emphysema (OR 3.73 [1.07–13.06]), which were all significantly associated with CAC > 100 in the intermediate unhealthy lifestyle group (Figure 4, Supplementary Table S8).

## 4. Discussion

In this study, we investigated the association between emphysema and CAC score in an urban Chinese general population cohort of 871 participants enrolled in 2017, considering the potential modifying effects of lifestyle factors. Unfavorable lifestyle factors, including smoking and alcohol consumption, were more prevalent in individuals with higher CAC scores. Paraseptal and severe emphysema were associated with higher CAC scores, even after adjusting for key confounders such as age, sex, education level, and cardiovascular risk factors. The associations remained significant after adjusting for unfavorable lifestyles (either as individual factors or as a cumulative number). Additionally, stratified analyses demonstrated that the strongest associations, particularly for paraseptal, moderate, and severe emphysema with CAC > 100, occurred in the intermediate unhealthy lifestyle group (i.e., participants with 2–3 unhealthy lifestyle factors), but these differences were not significant.

The observed emphysema prevalence of 62.2% exceeds that reported in Western general population cohorts (20.1–30.7%) [26,27]. This discrepancy may result from the inclusion of subtle emphysema (trace or more), which often represents asymptomatic structural changes underreported in clinical practice [28]. Additionally, the older age distribution (median age: 62) indicated a higher likelihood of subclinical structural lung changes and cumulative environmental damage [28]. Notably, the large female proportion (54.5%) could also contribute to the observed findings. Exposure to cooking fumes and biomass fuels, which is common in the Chinese population, has been recognized as an important risk factor for emphysema among non-smoking women [29]. Although this composite exposure showed no significant difference across CAC categories in our study, lung damage from cooking fumes may occur earlier than changes in coronary arteries. Future studies with more specific metrics are needed to better distinguish these effects. The observed distribution, characterized by a predominance of mild centrilobular emphysema and a relatively low proportion of paraseptal emphysema at 6.0%, aligns with findings from other east Asian screening cohorts and reflects the specific phenotype distribution of this demographic [30].

Two previous studies published in 2018 and 2021 have reported mixed results regarding the association between emphysema and calcium score [10,12]. Consistent with our study, CAC was assessed using calcium score on non-ECG-gated chest CT, which can introduce potential deviations in absolute Agatston scores due to motion artifacts. However, non-triggered chest CT has been shown to agree well with cardiac CT for CAC detection and risk stratification and is suitable for large-scale screening [31,32]. Our findings showed that paraseptal and severe emphysema was linked to a higher probability of coronary artery calcification. This supports the hypothesis that vascular alterations in the lungs may reflect or contribute to systemic atherosclerosis, potentially through shared mechanisms of “vascular–alveolar interface damage,” as paraseptal emphysema is more frequently associated with systemic inflammation and alveolar-capillary destruction compared to other types [33,34]. Nevertheless, Bhatt et al. found an independent association between centrilobular emphysema and CAC [10]. This discrepancy may be attributed to differences in study populations, as their research focused on individuals with varying degrees of COPD severity, whereas in our urban population cohort, most participants had mild emphysema. Moreover, the stronger association observed for severe emphysema indicates that greater lung destruction correlates with higher coronary calcification severity.

The cardiovascular impact of emphysema seems to be driven by specific phenotypes, since we only found significant associations for paraseptal and severe emphysema and CAC score, which remained significant even after adjusting for lifestyle factors. Unfavorable lifestyle factors, including smoking and alcohol consumption, were more frequently observed in participants with higher CAC scores [35,36,37]. While these factors are well-established contributors to cardiovascular disease, our study found no evidence that these factors modify the association between emphysema and CAC. This suggests that the link between emphysema and CAC may be driven by biological mechanisms independent of traditional behavioral risk factors. Possible mechanisms may include systemic inflammation originating from lung disease, oxidative stress, vascular endothelial dysfunction, and hypoxia-induced vascular remodeling, all of which have been implicated in both emphysema progression and atherosclerotic processes [38,39,40]. Additionally, shared genetic susceptibility may also play a role [41,42]. These findings from previous studies support the hypothesis of overlapping pathophysiological pathways between chronic lung disease and subclinical cardiovascular disease, highlighting the need for further research to better understand these mechanisms. Eventually, this may support earlier identification of individuals at risk, allowing timely implementation of lifestyle modifications, preventive therapies, or structured monitoring.

Although the association between emphysema and CAC score was not different between the presence and absence of unfavorable lifestyle factors, the stratified analyses did uncover several notable patterns. Unfavorable lifestyle was constructed using composite variables with six unhealthy lifestyle factors. Therefore, an intermediate unfavorable lifestyle should be interpreted as a measure of cumulative risk burden rather than a precise biological threshold. Considering the multiple stratified comparisons performed, these subgroup analyses were intended to explore potential associations and should be interpreted cautiously. For example, paraseptal emphysema was associated with CAC in seemingly lower-risk groups (those with healthy diets, higher physical activity, or no environmental exposures), which needs further investigation. Several potential explanations can be given for this observation. First, in the subgroup with less healthy dietary habits, the associations between paraseptal emphysema and CAC were attenuated and not statistically significant, possibly due to a limited sample size in this group. Secondly, there may be unmeasured confounding factors in these subgroups, or finally, paraseptal emphysema in these individuals may represent a distinct phenotype with particularly strong systemic inflammation effects [43,44]. In the intermediate unhealthy lifestyle group, the association between emphysema severity and advanced CAC scores may support the concept of cumulative risk exposure [45]. This finding suggests that while emphysema may independently increase cardiovascular risk, the coexistence of multiple unhealthy behaviors could potentially accelerate atherogenesis through synergistic mechanisms [45,46,47]. However, having four to six unhealthy lifestyle factors did not lead to higher CAC scores in individuals with emphysema. In addition to the small sample size in this extreme group, competing mechanisms could also play a role. For instance, certain behaviors such as alcohol consumption may have mixed effects on cardiovascular risk, potentially weakening the overall association in the high-risk group. As small sample sizes in certain subgroups resulted in unstable estimates with wide confidence intervals, these subgroup analyses were considered exploratory. Further research is needed to explore potential gene–environment interactions that may modify this relationship.

This study has several strengths, including comprehensive adjustment for confounders (age, sex, comorbidities, and lifestyle factors) and detailed characterization of emphysema subtypes that revealed differential associations with CAC (particularly for paraseptal and severe emphysema). Additionally, the short interval between the questionnaire and LDCT ensured consistency between lifestyle data and CT findings, supporting the accuracy of the cross-sectional analysis. Despite the strengths of our study, there are also limitations that should be considered. The cross-sectional design of our study precludes causal inference and the recruitment of older participants from a single urban region may limit generalizability. In addition, since the data was generated in 2017 before the COVID-19 pandemic, it may not fully reflect the health status of the population to date. In addition, self-report bias cannot be excluded, as lifestyle factors and history of cardiovascular disease were obtained from self-reported questionnaires. Furthermore, selection bias may have been introduced as participant recruitment was on a voluntary basis. Although we relied on CT-based assessment of emphysema, the reproducibility and subtype categorization and severity were not specifically quantified and validated. While it may not fully capture functional measures of disease severity that could provide additional information, it may also miss early disease. Finally, the relatively small sample size in some subgroup analyses may have limited our power to detect significant interaction effects. Despite these limitations, the associations between specific emphysema subtypes and CAC, independent of traditional risk factors, warrant further investigation into underlying mechanisms and potential clinical applications.

Integrating emphysema assessment into routine LDCT interpretation and considering concurrent CAC evaluation could help identify individuals at elevated risk for both pulmonary and cardiovascular disease. While emphysema may signal subclinical atherosclerosis, vascular calcification itself might also contribute to or reflect pulmonary tissue destruction [48]. As LDCT is already widely applied in lung cancer screening, leveraging incidental findings such as emphysema and CAC offers a cost-effective opportunity to improve early detection and risk stratification for comorbid conditions like atherosclerosis and COPD. Moreover, identifying individuals with intermediate lifestyle risk could support more personalized and preventive healthcare strategies [49,50].

## 5. Conclusions

In this study, we found that paraseptal and severe emphysema were associated with higher calcium scores, independent of key confounders and unfavorable lifestyle factors. Stratified analyses demonstrated that associations occurred in the intermediate unhealthy lifestyle subgroup, even though there was no significant difference between the groups. These findings underscore the importance of thorough cardiovascular risk assessment in emphysema individuals. Further research is required to suggest the role of emphysema as an early marker of elevated cardiovascular risk and to explore its potential utility in guiding targeted cardiovascular screening strategies.

## Figures and Tables

**Table 1:** Emphysema severity categories by type and extent.

Severity	Centrilobular Emphysema	Paraseptal Emphysema	Panlobular Emphysema
**Mild**	Trace to mild: lucencies occupying 5% of a lung zone	Trace to mild: >5 small (<1 cm) lucencies	Mild: involving a segment
**Moderate**	Moderate: >5% of a lung zone	Moderate: mainly large (>1 cm) juxtapleural cyst-like lucencies	Moderate: more than segmental
**Severe**	Confluent and advanced	Substantial: multiple groups of juxtapleural lucencies	Severe: entire lobe involved

**Table 2:** Demographic characteristics of participants, overall and stratified by CAC group (0, 1–100, >100).

Characteristics	Overall (n = 871, 100%)	CAC Group			*p*-Value
		Score 0 (n = 536, 61.5%)	Score 1–100 (n = 221, 25.4%)	Score > 100 (n = 114, 13.1%)	
**Age (median, range)**	62 (40–76)	60 (40–74)	64 (40–76)	65 (48–74)	<0.001 *
**Sex**					**<0.001**
**Male**	396 (45.5%)	172 (32.1%)	141 (63.8%)	83 (72.8%)	
**Female**	475 (54.5%)	364 (67.9%)	80 (36.2%)	31 (27.2%)	
**Education level ^&^**					**0.02**
**Low**	343 (39.4%)	199 (37.1%)	86 (38.9%)	58 (50.9%)	
**High**	528 (60.6%)	337 (62.9%)	135 (61.1%)	56 (49.1%)	
**Emphysema presence**					**<0.001**
**No**	329 (37.8%)	236 (44.0%)	68 (30.8%)	25 (21.9%)	
**Yes**	542 (62.2%)	300 (56.0%)	153 (69.2%)	89 (78.1%)	
**Emphysema type**					
**Centrilobular**	334 (38.3%)	194 (36.2%)	91 (41.2%)	49 (43.0%)	0.54
**Paraseptal**	52 (6.0%)	27 (5.0%)	15 (6.8%)	10 (8.8%)	
**Mixed type**	156 (17.9%)	79 (14.7%)	47 (21.3%)	30 (26.2%)	
**Emphysema severity**					
**Mild**	482 (88.9%)	277 (51.7%)	134 (60.6%)	71 (62.3%)	**<0.001**
**Moderate**	23 (4.2%)	9 (1.7%)	9 (4.1%)	5 (4.4%)	
**Severe**	37 (6.8%)	14 (2.6%)	10 (4.5%)	13 (11.3%)	
**Diabetes**					**<0.001**
**No**	751 (86.2%)	485 (90.5%)	178 (80.5%)	88 (77.2%)	
**Yes**	120 (13.8%)	51 (9.5%)	43 (19.5%)	26 (22.8%)	
**Hypertension**					**<0.001**
**No**	607 (69.7%)	412 (76.9%)	126 (57.0%)	69 (60.5%)	
**Yes**	264 (30.3%)	124 (23.1%)	95 (43.0%)	45 (39.5%)	
**Hyperlipidemia**					0.79
**No**	757 (86.9%)	463 (86.4%)	195 (88.2%)	99 (86.8%)	
**Yes**	114 (13.1%)	73 (13.6%)	26 (11.8%)	15 (13.2%)	

Note: * Using the Kruskal–Wallis H test. Mixed type of emphysema combines the mixed type and panlobular subtype. **^&^** Participants with less than college education are defined as having a low education level, while those with a college education or above are defined as having a high education level.

**Table 3:** Unfavorable lifestyles of participants, overall and stratified by CAC group (0, 1–100, >100).

Characteristics	Overall (n = 871, 100%)	CAC Group			*p* Value
		Score 0 (n = 536, 61.5%)	Score 1–100 (n = 221, 25.4%)	Score > 100 (n = 114, 13.1%)	
**Smoking**					**<0.001**
**Never smoking**	571 (65.6%)	403 (75.2%)	121 (54.8%)	47 (41.2%)	
**Current/former smoking**	300 (34.4%)	133 (24.8%)	100 (45.2%)	67 (58.8%)	
**Unfavorable BMI**					
**No**	368 (42.3%)	234 (43.7%)	85 (38.5%)	49 (43.0%)	0.42
**Yes**	503 (57.7%)	302 (56.3%)	136 (61.5%)	65 (57.0%)	
**Less healthy dietary habits**					0.17
**No**	654 (75.1%)	413 (77.1%)	162 (73.3%)	79 (69.3%)	
**Yes**	217 (24.9%)	123 (22.9%)	59 (26.7%)	35 (30.7%)	
**Low physical activity**					0.24
**No**	717 (82.3%)	432 (80.6%)	188 (85.1%)	97 (85.1%)	
**Yes**	154 (17.7%)	104 (19.4%)	33 (14.9%)	17 (14.9%)	
**Alcohol consumption**					**<0.001**
**No**	683 (78.4%)	446 (83.2%)	160 (72.4%)	77 (67.5%)	
**Yes**	188 (21.6%)	90 (16.8%)	61 (27.6%)	37 (32.5%)	
**Environmental exposure**					0.65
**No**	313 (35.9%)	193 (36.0%)	83 (37.6%)	37 (32.5%)	
**Yes**	558 (64.1%)	343 (64.0%)	138 (62.4%)	77 (67.5%)	
**No. of unfavorable lifestyle factors**					**<0.001**
**0–1 (favorable lifestyle)**	283 (32.5%)	194 (36.2%)	64 (29.0%)	25 (21.9%)	
**2–3 (intermediate lifestyle)**	429 (49.3%)	265 (49.4%)	108 (48.9%)	56 (49.1%)	
**4–6 (unfavorable lifestyle)**	159 (18.3%)	77 (14.4%)	49 (22.1%)	33 (28.9%)	

## Data Availability

The original contributions presented in this study are included in the article. Further inquiries can be directed to the corresponding author.

## Supplementary Materials

The following supporting information can be downloaded at: https://www.mdpi.com/article/10.3390/healthcare14060736/s1, Figure S1. Directed Acyclic Graph for the relationship between emphysema and calcium score. Table S1. The translation of unfavorable lifestyle variables used in Table 3 according to the questionnaire. Table S2. Multinominal logistic regression analysis for associations between emphysema and different CAC group. Table S3. Multinominal logistic regression analysis for associations between emphysema and CAC adjusted by unfavourable lifestyle factors. Table S4. Multinominal logistic regression analysis for associations between emphysema and CAC adjusted by number of unfavourable lifestyle factors. Table S5. Multinominal logistic regression and interaction analysis for associations between emphysema presence and different CAC group stratified by each lifestyle. Table S6. Multinominal logistic regression and interaction analysis for associations between emphysema type and different CAC group stratified by each lifestyle. Table S7. Multinominal logistic regression and interaction analysis for associations between emphysema severity and different CAC group stratified by each lifestyle. Table S8. Multinominal logistic regression and interaction analysis for associations between emphysema and different CAC group stratified by each number of unhealthy lifestyles.

## Abbreviations

The following abbreviations are used in this manuscript:

LDCT	Low-dose CT
LCS	Lung cancer screening
BMI	Body mass index
MinIP	Minimum intensity projection
MPR	Multiplanar reconstruction
ORs	Odds ratios
CIs	Confidence intervals
COPD	Obstructive pulmonary disease
CVD	Cardiovascular disease
