# Supplementary material for: Association Between Emphysema and Coronary Artery Calcium on Low-Dose CT in Urban Chinese Adults: Does Lifestyle Matter?
Source: Healthcare (Basel). 2026 Mar 13;14(6):736. doi: 10.3390/healthcare14060736 (PMC13027018; doi:10.3390/healthcare14060736)
Supplement: Supplementary file 1 [file healthcare-14-00736-s001.zip › healthcare-4147019-supplementary.pdf]

Supplementary Table S1. The translation of unfavorable lifestyle variables used in Tables 3 according to the questionnaire.

Characteristic	Description of unfavorable lifestyle variables	Questionnaire
Smoking	Current or former smokers	Have you ever sucked at least 1 cigarette a day and lasted for 6 months or more?<br>□ (1) never suck (2) Yes, is still sucking (3) have ever smoked but quit smoking for□□years
Unfavorable BMI	Having BMI <18.5 or ≥24.0kg/cm²	-
Less healthy dietary habits	Having any of the three dietary habits (not eating fresh fruits daily, not eating vegetables daily, or eating red meat daily)	In the last year, the following foods which you ate every day? (Multiple choice)<br>(1) fresh vegetables □ (2) fresh fruits □ (3) cereals □ (4) fresh meat □ (5) eggs □ (6) none of the above □
Low physical activity	Low or moderate level of physical activity, which including sedentary behaviour, mode of transportation and exercise types.<br><br>Scoring rules for total physical activity scoring:<br>1.High level: requires at least two high-benefit components from these three variables, but low-benefit components included;<br>2. Moderate Level: the combination of low and high-benefit components or two high-benefit components with one low-benefit component.<br>3. Low Level: at least two low-benefit components or including only one low-benefit component but no high-benefit components.	The level of each variable were shown as follows:<br>1. Sedentary Behavior<br>☐ Low level: Sitting time less than 3 hours/day.<br>☐ Moderate level: Sitting time between 3-6 hours/day.<br>☐ High level: Sitting time more than 7 hours/day.<br><br>2. Mode of Transportation<br>☐ High activity level: Primarily relies on walking or cycling for daily activities.<br>☐ Moderate activity level: Uses public transportation combined with walking.<br>☐ Low activity level: Primarily relies on private cars with minimal walking.<br><br>3. Exercise Types<br>☐ High activity level: Engages in high-intensity or moderate-intensity exercise weekly, such as running, swimming, or playing sports.<br>☐ Moderate activity level: Participates in at least one moderate-intensity exercise weekly, such as brisk walking or yoga.

		☐ Low activity level: not exercise weekly.
Alcohol consumption	Drinking alcohol or beer more than 3 times/week.	In the last year, the following drink which you took at least 3 times a week? (Multiple choice)<br>(1) alcohol □ (2) beer □ (3) fresh milk □ (4) tea □ (5) coffee □ (6) none of the above □
Environmental exposures	Having any of the situation (more cooking fume, building for residence or work located along the street or exposed to toxic or harmful substances for three months or longer).	The questions about environmental exposures in the questionnaire were as follows:<br>1. In the past 10 years, the situation of smoke during your cooking:<br>□no smoke □ little □ more □ many.<br>2. For the past 10 years, have you been exposed to toxic substances in work place for 3 months or more?<br>□ No □ Yes, please specify (multiple choice):<br>□ Asbestos □ Cadmium □ Nickel □ Arsenic □ Radon □ Vinyl chloride □ X-ray □ Other<br>3. In the past 10 years, has your residential building or workplace office building been located on a main street, or within 20 meters of a busy motor vehicle road?<br>☐ Yes; ☐ No

Note: Unfavorable lifestyle variables indicate the presence of having any six unhealthy lifestyle factors (smoking, unhealthy BMI, less healthy dietary habits, low physical activity, alcohol consumption and environmental exposure).

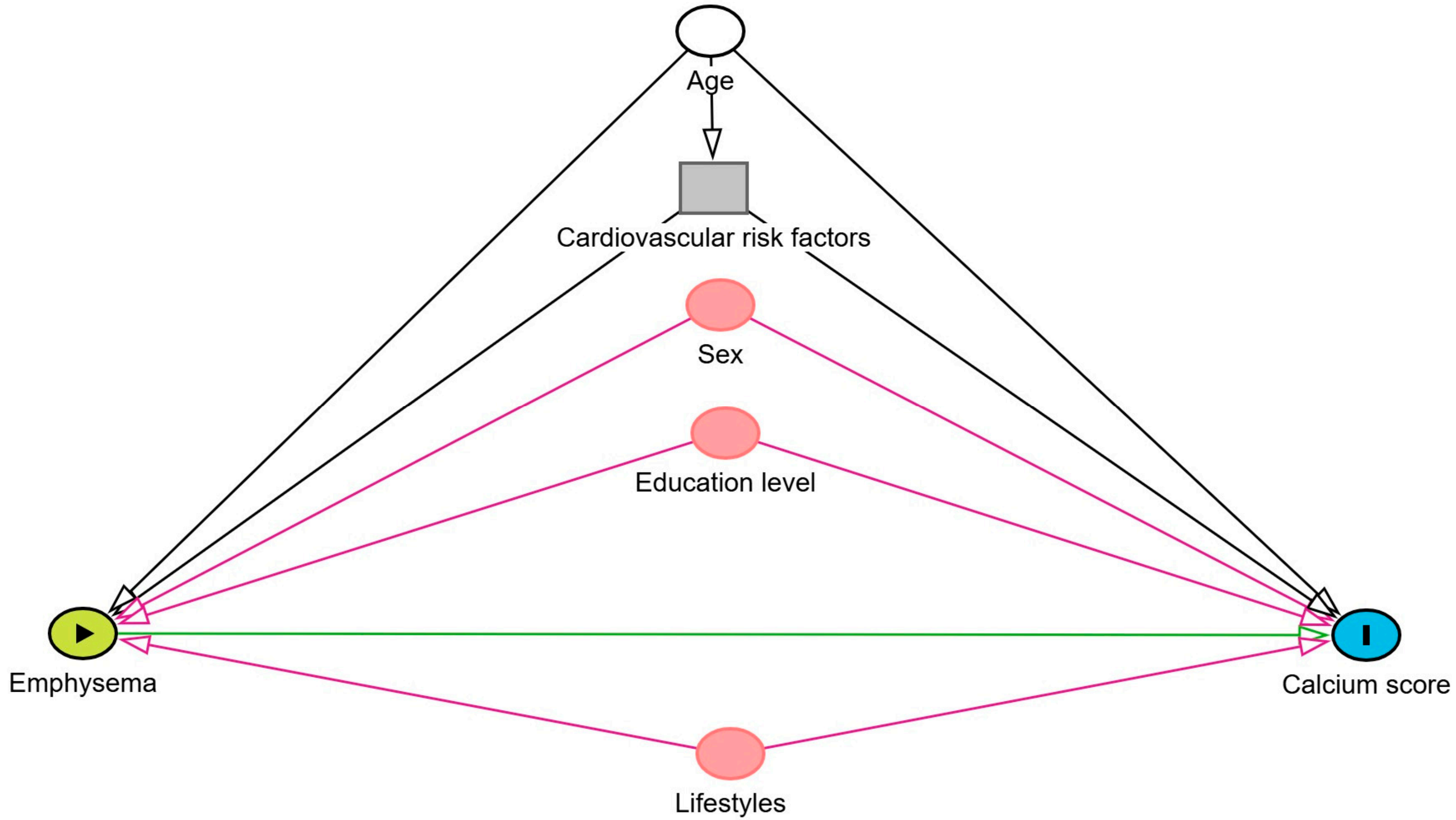

Supplementary Figure S1. Directed Acyclic Graph for the relationship between emphysema and calcium score. Analyses were stratified by each lifestyle separately (i.e. smoking, BMI, diet, physical activity, alcohol, and environmental exposure) and adjusted for the other lifestyles.

Supplementary Table S2. Multinominal logistic regression analysis for associations between emphysema and different CAC group.

Emphysema	CAC 1-100		CAC >100	
	OR (95% CI)	*P* value	OR (95% CI)	*P* value
**Emphysema presence**				
**No**	Ref.		Ref.	
**Yes**	0.95 (0.65-1.38)	0.77	0.70 (0.42-1.19)	0.19
**Emphysema type**				
**No type**	Ref.		Ref.	
**Centrilobular**	0.99 (0.66-1.50)	0.97	1.20 (0.72-2.01)	0.48
**Paraseptal**	1.86 (0.87-3.96)	0.11	**2.94 (1.26-6.84)**	**0.01**
**Mixed type**	0.99 (0.59-1.65)	0.97	1.33 (0.72-2.44)	0.36
**Emphysema severity**				
**No**	Ref.		Ref.	
**Mild**	1.02 (0.70-1.50)	0.91	1.27 (0.75-2.17)	0.37
**Moderate**	2.03 (0.74-5.60)	0.17	2.60 (0.74-9.12)	0.14
**Severe**	1.15 (0.45-2.96)	0.77	**3.26 (1.24-8.57)**	**0.02**

Note: All models were adjusted by age, sex, education level, cardiovascular risk factors (i.e. diabetes, hypertension, hyperlipidemia).

Supplementary Table S3. Multinominal logistic regression analysis for associations between emphysema and CAC adjusted by unfavorable lifestyle factors.

Emphysema	CAC 1-100		CAC >100	
	OR (95% CI)	*P* value	OR (95% CI)	*P* value
**Emphysema presence**				
**No**	Ref.		Ref.	
**Yes**	0.95 (0.65-1.40)	0.80	0.78 (0.46-1.34)	0.37
**Emphysema type**				
**No type**	Ref.		Ref.	
**Centrilobular**	0.99 (0.65-1.45)	0.96	1.18 (0.66-2.10)	0.58
**Paraseptal**	1.81 (0.84-3.90)	0.13	**2.84 (1.09-7.38)**	**0.03**
**Mixed type**	0.99 (0.59-1.69)	0.99	1.17 (0.59-2.31)	0.65
**Emphysema severity**				
**No**	Ref.		Ref.	
**Mild**	1.02 (0.69-1.51)	0.91	1.18 (0.69-2.03)	0.56
**Moderate**	1.99 (0.71-5.57)	0.19	2.09 (0.57-7.58)	0.26
**Severe**	1.13 (0.43-2.95)	0.80	**2.75 (1.02-7.42)**	**0.05**

Note: All models were adjusted by age, sex, cardiovascular risk factors (i.e. diabetes, hypertension, hyperlipidemia) and unfavorable lifestyle (pack-years of smoking, unhealthy BMI, less healthy dietary habits, low physical activity, alcohol consumption and environmental exposure).

Supplementary Table S4. Multinominal logistic regression analysis for associations between emphysema and CAC adjusted by number of unfavorable lifestyle factors.

**Emphysema**	**CAC 1-100**		**CAC >100**	
	**OR (95% CI)**	***P* value**	**OR (95% CI)**	***P* value**
**Emphysema presence**				
**No**	Ref.		Ref.	
**Yes**	0.96 (0.66-1.39)	0.82	0.71 (0.42-1.21)	0.21
**Emphysema type**				
**No type**	Ref.		Ref.	
**Centrilobular**	0.98 (0.65-1.48)	0.93	1.25 (0.71-2.21)	0.44
**Paraseptal**	1.83 (0.86-3.89)	0.12	**3.17 (1.24-8.09)**	**0.02**
**Mixed type**	0.98 (0.59-1.64)	0.95	1.33 (0.69-2.57)	0.40
**Emphysema severity**				
**No**	Ref.		Ref.	
**Mild**	0.81 (0.53-1.23)	0.32	1.26 (0.74-2.15)	0.40
**Moderate**	2.00 (0.72-5.54)	0.18	2.45 (0.69-8.67)	0.17
**Severe**	1.15 (0.45-2.93)	0.78	**3.21 (1.22-8.46)**	**0.02**

Note: All models were adjusted by age, sex, cardiovascular risk factors (i.e. diabetes, hypertension, hyperlipidemia) and number of unfavorable lifestyles.

Supplementary Table S5. Multinominal interaction logistic regression analysis for associations between emphysema presence and different CAC group stratified by each lifestyle.

Subgroups	Unhealthy lifestyle												P for interaction analysis
	No presence						Presence						
	CAC 1-100			CAC >100			CAC 1-100			CAC >100			
	N	OR (95% CI)	P value	N	OR (95% CI)	P value	N	OR (95% CI)	P value	N	OR (95% CI)	P value	
**Smoking**	73	1.31 (0.85-2.02)	0.22	30	1.25 (0.68-2.29)	0.47	80	0.97 (0.48-1.95)	0.93	59	1.17 (0.51-2.69)	0.71	0.22
**Unfavorable BMI**	65	1.73 (0.93-3.21)	0.08	39	1.04 (0.49-2.20)	0.92	87	1.01 (0.63-1.60)	0.99	50	1.34 (0.71-2.55)	0.37	0.49
**Less healthy dietary habits**	111	1.33 (0.88-2.01)	0.17	59	1.41 (0.81-2.44)	0.22	42	0.80 (0.36-1.78)	0.58	30	0.87 (0.30-2.53)	0.79	0.63
**Low physical activity**	131	1.08 (0.72-1.62)	0.71	77	1.36 (0.79-2.34)	0.27	22	1.85 (0.74-4.64)	0.19	12	0.50 (0.14-1.78)	0.28	0.33
**Alcohol consumption**	104	1.15 (0.77-1.73)	0.49	59	1.21 (0.70-2.10)	0.49	49	1.83 (0.74-4.51)	0.19	30	1.91 (0.66-5.54)	0.23	0.36
**Environmental exposure**	55	1.10 (0.61-2.01)	0.75	31	2.04 (0.79-5.29)	0.14	98	1.36 (0.85-2.17)	0.20	58	1.13 (0.64-2.02)	0.67	0.34

Note: All models were adjusted by age, sex, education level, cardiovascular risk factors (i.e. diabetes, hypertension, hyperlipidemia) and adjusted for other lifestyles. N indicates the number of participants with emphysema presence in CACS level (1–100 or >100).

Supplementary Table S6. Multinominal logistic regression analysis for associations between emphysema type and different CAC group stratified by each lifestyle.

Subgroups	Emphysema type	Unhealthy lifestyle												P for interaction analysis
		No presence						Presence						
		CAC 1-100			CAC >100			CAC 1-100			CAC >100			
		N	OR (95% CI)	P value	N	OR (95% CI)	P value	N	OR (95% CI)	P value	N	OR (95% CI)	P value	
**Smoking**	**Centrilobular**	56	1.41 (0.89-2.23)	0.14	22	1.24 (0.64-2.38)	0.52	35	0.70 (0.32-1.51)	0.36	27	0.88 (0.36-2.17)	0.78	0.06
	**Paraseptal**	6	1.61 (0.61-4.23)	0.33	2	1.59 (0.40-6.42)	0.51	9	2.74 (0.81-9.22)	0.10	8	**4.41 (1.16-16.78)**	**0.03**	0.63
	**Mixed type**	11	0.84 (0.39-1.81)	0.65	6	1.13 (0.44-2.95)	0.80	36	1.13 (0.50-2.53)	0.77	24	1.16 (0.45-3.01)	0.76	0.81
**Unfavorable BMI**	**Centrilobular**	38	1.70 (0.88-3.28)	0.11	19	0.93 (0.41-2.11)	0.87	53	0.91 (0.54-1.51)	0.70	30	1.20 (0.60-2.41)	0.60	0.37
	**Paraseptal**	6	2.07 (0.62-6.90)	0.24	4	2.61 (0.69-9.91)	0.16	9	1.86 (0.76-4.55)	0.17	6	2.83 (0.92-8.74)	0.07	0.52
	**Mixed type**	22	1.67 (0.74-3.77)	0.22	16	0.97 (0.37-2.50)	0.94	25	0.98 (0.50-1.93)	0.96	14	1.23 (0.52-2.91)	0.64	0.93
**Less healthy dietary habits**	**Centrilobular**	67	1.30 (0.83-2.03)	0.26	32	1.26 (0.69-2.30)	0.45	24	0.74 (0.32-1.72)	0.48	17	0.81 (0.26-2.46)	0.70	0.19
	**Paraseptal**	12	**2.42 (1.00-5.83)**	**0.05**	7	**3.01 (1.00-9.04)**	**0.05**	3	0.79 (0.18-3.42)	0.75	3	**2.81 (1.08-7.30)**	**0.03**	0.32
	**Mixed type**	32	1.09 (0.61-1.94)	0.78	20	1.22 (0.59-2.52)	0.59	15	1.06 (0.35-3.20)	0.92	10	1.17 (0.59-2.31)	0.66	0.97
**Low physical activity**	**Centrilobular**	77	0.98 (0.63-1.51)	0.91	46	1.28 (0.72-2.28)	0.41	14	2.62 (0.96-7.13)	0.06	3	0.26 (0.05-1.32)	0.11	0.26
	**Paraseptal**	13	1.83 (0.84-3.98)	0.13	8	2.71 (0.94-7.81)	0.07	2	3.11 (0.39-24.51)	0.28	2	2.62 (0.29-23.77)	0.39	0.25
	**Mixed type**	41	1.11 (0.64-1.95)	0.71	23	1.19 (0.58-2.41)	0.64	6	0.73 (0.20-2.73)	0.64	7	0.64 (0.13-3.24)	0.59	0.47
**Alcohol consumption**	**Centrilobular**	70	1.16 (0.75-1.79)	0.51	35	1.15 (0.64-2.09)	0.64	21	1.45 (0.55-3.84)	0.45	14	1.43 (0.45-4.59)	0.55	0.82
	**Paraseptal**	11	1.81 (0.82-3.97)	0.14	7	**3.00 (1.01-8.94)**	**0.05**	4	3.30 (0.55-19.88)	0.19	3	5.94 (0.90-39.15)	0.06	0.88
	**Mixed type**	23	0.89 (0.48-1.64)	0.71	17	1.01 (0.47-2.18)	0.97	24	2.34 (0.81-6.80)	0.12	13	2.21 (0.65-7.54)	0.21	0.08
**Environmental exposure**	**Centrilobular**	36	1.07 (0.57-2.04)	0.83	18	1.76 (0.64-4.82)	0.28	55	1.28 (0.78-2.13)	0.33	31	1.01 (0.54-1.89)	0.98	0.51
	**Paraseptal**	7	1.56 (0.52-4.65)	0.42	**5**	**5.63 (1.30-24.35)**	**0.02**	8	2.38 (0.91-6.25)	0.08	5	2.14 (0.70-6.54)	0.18	0.37
	**Mixed type**	12	0.95 (0.39-2.34)	0.91	8	1.74 (0.47-6.43)	0.40	35	1.30 (0.70-2.43)	0.40	22	1.18 (0.56-2.47)	0.66	0.99

Note: All models were adjusted by age, sex, education level, cardiovascular risk factors (i.e. diabetes, hypertension, hyperlipidemia) and other lifestyles. N indicates the number of participants with emphysema type in CACS level (1–100 or >100).

Supplementary Table S7. Multinominal logistic regression analysis for associations between emphysema severity and different CAC group stratified by each lifestyle.

Subgroups	Emphysema severity	Unhealthy lifestyle												P for interaction analysis
		No presence						Presence						
		CAC 1-100			CAC >100			CAC 1-100			CAC >100			
		N	OR (95% CI)	P value	N	OR (95% CI)	P value	N	OR (95% CI)	P value	N	OR (95% CI)	P value	
**Smoking**	**Mild**	69	1.32 (0.85-2.04)	0.21	28	1.24 (0.67-2.29)	0.49	65	0.91 (0.45-1.85)	0.79	43	0.98 (0.42-2.30)	0.97	0.11
	**Moderate**	1	0.61 (0.06-5.72)	0.66	0	0	0	8	3.08 (0.74-12.81)	0.12	5	3.95 (0.80-19.54)	0.09	0.44
	**Severe**	3	1.96 (0.36-10.80)	0.44	2	3.18 (0.44-23.02)	0.25	7	0.98 (0.28-3.52)	0.98	11	3.13 (0.88-11.10)	0.08	0.46
**Unfavorable BMI**	**Mild**	57	1.73 (0.93-3.23)	0.09	29	0.96 (0.45-2.05)	0.91	77	0.97 (0.61-1.55)	0.89	42	1.25 (0.65-2.40)	0.50	0.27
	**Moderate**	6	3.69 (0.88-15.54)	0.08	1	0.88 (0.13-6.08)	0.89	3	1.79 (0.36-8.90)	0.48	4	**5.46 (1.00-30.42)**	**0.05**	0.86
	**Severe**	3	0.80 (0.17-3.85)	0.78	9	2.75 (0.74-10.24)	0.13	7	1.98 (0.54-7.32)	0.30	4	3.70 (0.93-16.51)	0.09	0.54
**Less healthy dietary habits**	**Mild**	101	0.77 (0.44-1.35)	0.37	48	1.02 (0.56-1.86)	0.95	33	0.76 (0.34-1.73)	0.52	23	0.76 (0.26-2.28)	0.63	0.20
	**Moderate**	3	0.29 (0.07-1.27)	0.10	4	0.37 (0.07-1.86)	0.23	6	3.30 (0.61-18.01)	0.17	1	1.08 (0.11-10.95)	0.95	0.92
	**Severe**	7	2.94 (0.81-10.61)	0.10	7	0.51 (0.16-1.69)	0.27	3	0.50 (0.09-2.71)	0.42	6	**2.83 (1.05-7.67)**	**0.04**	0.86
**Low physical activity**	**Mild**	115	1.05 (0.70-1.57)	0.82	64	1.26 (0.73-2.18)	0.41	19	1.92 (0.76-4.84)	0.17	7	0.43 (0.12-1.61)	0.21	0.41
	**Moderate**	8	2.64 (0.81-8.56)	0.11	5	3.62 (0.87-15.00)	0.08	1	0.69 (0.05-10.44)	0.79	0	0	0	0.22
	**Severe**	8	1.01 (0.34-2.98)	0.99	8	2.34 (0.77-7.10)	0.13	2	1.47 (0.17-14.64)	0.73	5	1.95 (0.24-15.66)	0.53	0.89
**Alcohol consumption**	**Mild**	94	1.13 (0.75-1.70)	0.55	49	1.09 (0.62-1.92)	0.76	40	1.79 (0.72-4.44)	0.21	22	1.74 (0.59-5.14)	0.31	0.67
	**Moderate**	4	1.33 (0.36-4.98)	0.67	2	1.88 (0.38-9.22)	0.44	5	6.67 (0.87-51.32)	0.07	3	5.73 (0.55-59.50)	0.14	0.13
	**Severe**	6	1.92 (0.54-6.86)	0.31	8	**5.30 (1.48-19.04)**	**0.01**	4	1.14 (0.23-5.70)	0.87	5	2.87 (0.54-15.36)	0.22	0.70
**Environmental exposure**	**Mild**	49	1.09 (0.60-2.00)	0.78	25	1.86 (0.70-4.89)	0.21	85	1.31 (0.82-2.10)	0.26	46	1.04 (0.58-1.87)	0.89	0.53
	**Moderate**	2	0.87 (0.14-5.28)	0.88	2	3.86 (0.52-28.65)	0.19	7	**4.18 (1.10-15.87)**	**0.04**	3	2.62 (0.51-13.52)	0.25	0.94
	**Severe**	4	1.75 (0.36-8.38)	0.49	4	5.13 (0.89-29.58)	0.07	6	1.33 (0.40-4.47)	0.65	9	2.73 (0.84-8.89)	0.09	0.50

Note: All models were adjusted by age, sex, education level, cardiovascular risk factors (i.e. diabetes, hypertension, hyperlipidemia) and other lifestyles. N indicates the number of participants with emphysema severity in CACS level (1–100 or >100).

Supplementary Table S8. Multinominal logistic regression analysis for associations between emphysema and different CAC group stratified by each number of unhealthy lifestyles.

Emphysema	Unhealthy lifestyle																		P for interaction analysis
	0–1 (favorable lifestyle)						2–3 (intermediate lifestyle)						4–6 (unfavorable lifestyle)						
	CAC 1-100			CAC >100			CAC 1-100			CAC >100			CAC 1-100			CAC >100			
	N	OR (95% CI)	P value	N	OR (95% CI)	P value	N	OR (95% CI)	P value	N	OR (95% CI)	P value	N	OR (95% CI)	P value	N	OR (95% CI)	P value	
**Presence**	42	1.23 (0.65-2.31)	0.52	16	0.90 (0.37-2.19)	0.82	73	1.17 (0.70-1.94)	0.55	46	1.87 (0.95-3.68)	0.07	38	1.31(0.67-2.59)	0.88	27	0.92 (0.30-2.80)	0.88	0.59[a], 0.48[b]
**Centrilobular**	32	1.32 (0.67-2.59)	0.42	11	0.89 (0.34-2.32)	0.82	43	1.10 (0.63-1.91)	0.75	25	1.62 (0.77-3.39)	0.20	16	0.79 (0.28-2.21)	0.65	13	0.72 (0.21-2.46)	0.60	0.76[a], 0.25[b]
**Paraseptal**	5	1.48 (0.44-5.06)	0.53	1	0.78 (0.09-7.17)	0.83	6	2.14 (0.75-6.11)	0.16	**6**	**5.41 (1.70-17.22)**	**0.004**	4	2.35 (0.41-13.46)	0.34	3	1.73 (0.23-12.94)	0.59	0.31[a], 0.75[b]
**Mixed type**	5	0.78 (0.27-2.30)	0.66	4	0.97 (0.25-3.67)	0.96	24	1.06 (0.53-2.11)	0.87	15	1.57 (0.66-3.73)	0.31	18	1.42 (0.47-4.32)	0.54	11	1.15 (0.31-4.20)	0.84	0.73[a], 0.86[b]
**Mild**	41	1.26 (0.67-2.37)	0.48	15	0.90 (0.37-2.20)	0.82	63	1.12 (0.67-1.88)	0.66	36	1.64 (0.82-3.29)	0.16	30	0.98 (0.38-2.58)	0.97	20	0.75 (0.24-2.37)	0.63	0.73[a], 0.38[b]
**Moderate**	1	0.58 (0.06-5.94)	0.64	0	0	0	3	2.90 (0.52-16.07)	0.22	3	**9.64 (1.64-56.55)**	**0.01**	5	3.25 (0.51-20.62)	0.21	2	1.48 (0.14-15.47)	0.74	0.17[a], 0.46[b]
**Severe**	0	0	0	1	22.17 (0.21-2376.14)	0.19	7	1.40 (0.44-4.47)	0.57	7	**3.73 (1.07-13.06)**	**0.04**	3	0.84 (0.14-5.12)	0.85	5	1.99 (0.34-11.51)	0.44	0.49[a], 0.30[b]

Note: All models were adjusted by age, sex, education level, cardiovascular risk factors (i.e. diabetes, hypertension, hyperlipidemia). *P*-values for interaction were derived from models including interaction terms between emphysema presence/type/severity and each unhealthy lifestyle factor; a indicated the interaction between emphysema and intermediate lifestyle (2–3 unhealthy factors), and b indicated the interaction between emphysema and unfavorable lifestyle (4–6 unhealthy factors). N indicates the number of participants with emphysema presence/type/severity in CACS level (1–100 or >100).
